# PixelCut: A Unified Solution for Zero-Configuration 16S rRNA Trimming via Computer Vision

**DOI:** 10.3390/cimb47120968

**Published:** 2025-11-21

**Authors:** Dongin Kim, Woo Jin Kim, Hyun-Myung Woo, Hyundoo Jeong

**Affiliations:** 1 Department of Biomedical and Robotics Engineering, Incheon National University, Incheon 22012, Republic of Korea; gtp.atpase@gmail.com; 2 Department of Laboratory Medicine, EONE Laboratories, 291 Harmony-ro, Yeonsu-gu, Incheon 22014, Republic of Korea; oojinkim@hanmail.net

**Keywords:** microbiome, 16S rRNA sequencing, taxonomy profiling, web application, trimming position prediction

## Abstract

16S rRNA amplicon sequencing has been an effective method for profiling microbial taxonomy in microbiome research, as it offers lower per-sample costs and higher sample throughput than shotgun metagenomics. Although 16S rRNA sequencing offers clear advantages over shotgun sequencing, it depends on precise trimming of low-quality bases at the 3′ ends of reads. Given the widespread use of 16S rRNA amplicon sequencing, there is an increasing demand for analysis tools that can identify errors in the 3′ region of reads and remove erroneous bases. While various algorithms for predicting trim locations are widely employed, most are command-line standalone tools, which pose challenges for users with limited computational background or resources. Furthermore, in the absence of biological or experimental priors such as amplicon size, trim position predictions may be unreliable. Here, we introduce PixelCut, a fully automated trim-position prediction framework that requires no hyperparameters or prior biological information for accurate prediction. Unlike most available algorithms that operate on raw FASTQ data, PixelCut analyzes the per-base quality report generated by FastQC to infer trimming positions. Based on the recommended quality score threshold from the quality report, PixelCut inspects the quality scores across bases and automatically determines the recommended trim position using character recognition techniques based on computer vision. We have also developed a user-friendly web application to make the method accessible to those without programming expertise, while offering a command-line version for advanced users. Through comprehensive computer simulations, we show that PixelCut produces taxonomic profiling results that are consistent with those from popular trim-location prediction algorithms.

## 1. Introduction

The 16S ribosomal RNA (rRNA) gene is a component of the small subunit of prokaryotic ribosomes and contains both conserved and hypervariable regions. By amplifying and sequencing specific hypervariable regions (e.g., V1–V9), 16S rRNA amplicon sequencing is a targeted sequencing approach that amplifies and analyzes hypervariable regions of the 16S ribosomal RNA gene, a highly conserved genetic marker present in all prokaryotes. This technique enables taxonomic identification and profiling of bacterial and archaeal communities based on sequence variation in these hypervariable regions [1,2]. While shotgun metagenomic sequencing also enables comprehensive functional analysis such as metagenome assembly and pathway annotation, it requires high experimental resources so that its application can be limited to practical environments [3,4]. Because 16S rRNA amplicon sequencing typically requires fewer experimental and computational resources than shotgun metagenomic sequencing, it has become a popular starting point for large-scale or longitudinal microbiome studies [5,6].

To derive in-depth analytical results and accurately identify microbial communities through 16S rRNA amplicon sequencing, a number of analysis pipelines have been introduced. These pipelines are generally based on either OTU (operational taxonomic unit) clustering or ASV (amplicon sequence variant) inference. Although OTU-based pipelines such as QIIME1 [7] and MOTHUR [8] were once the standard approach to identifying microbial communities, ASV-based frameworks such as DADA2 [9] have been gaining popularity due to their ability to provide higher-resolution results and generate outputs without the need for reference sequences [10]. Both approaches typically benefit from merging paired-end reads to reconstruct full-length amplicons, which can improve the accuracy of taxonomic assignment. Hence, accurate prediction of trimming positions prior to merging is essential, as improper trimming can lead to merging failure or loss of informative sequences [11]. During the pre-processing step, careful trimming of paired-end reads can significantly improve downstream analytical accuracy, particularly in ASV-based workflows that rely on error modeling [12]. With the increasing demand for 16S rRNA amplicon sequencing, there is a growing need for tailored algorithms that improve the reliability and accuracy of analysis results. In particular, trimming algorithms capable of accurately identifying sequencing errors at the $3^{\prime }$ ends of reads and removing low-quality bases are essential for improving downstream performance.

To address this need, FIGARO has been introduced as an automated tool that analyzes the quality profiles of paired-end reads to determine optimal trimming parameters [13]. FIGARO is fully compatible with the DADA2 analysis pipeline and helps ensure sufficient read overlap while minimizing the inclusion of low-quality regions [13]. Despite its utility, FIGARO can be challenging to install and apply effectively, especially for users without a computational background or access to sufficient computing resources. Additionally, for users with limited biological expertise, interpreting quality profiles and setting appropriate trimming parameters, such as those related to amplicon size or primer sequences, can be non-trivial and error-prone. To bridge the gap between computational and biological users, we developed a user-friendly trimming algorithm, PixelCut, which automates parameter selection while minimizing the need for domain-specific knowledge. This approach aims to improve usability and reproducibility, making high-quality pre-processing more accessible to a broader range of researchers. PixelCut utilizes quality control (QC) reports that are typically generated alongside FASTQ sequencing results. It detects low-quality regions in the QC report using the OpenCV library and automatically identifies the precise trimming boundaries at the $3^{\prime }$ ends of reads. As a result, it eliminates the need for manual parameter tuning, which often requires biological expertise and can be time-consuming to optimize. Additionally, PixelCut is fully implemented as a web application, making 16S rRNA amplicon sequencing analysis more accessible to users with limited experience in command-line tools or computational resources. We also provide a standalone package for users who wish to integrate this fully automated pre-processing method into their own pipelines.

The Materials and Methods section details the data sources, pre-processing steps, and algorithmic framework used in the study. The Results section presents experimental setup and the performance analysis results. Finally, the Discussion summarizes the key contributions and highlights potential directions for future work.

## 2. Materials and Methods

**Figure 1:**
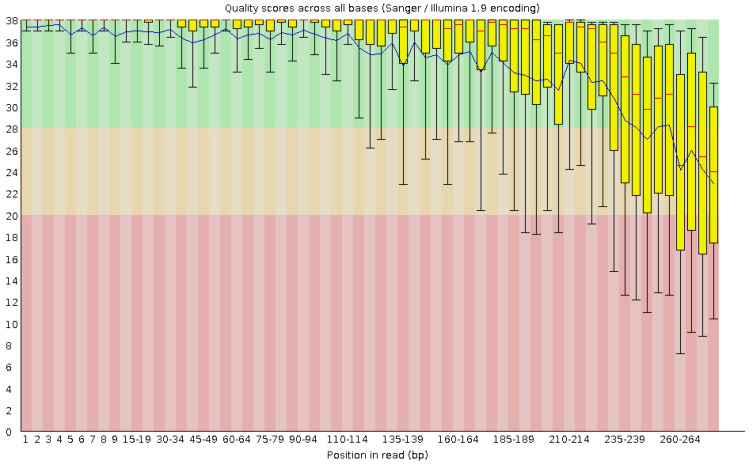
Example of a per−base sequence quality report generated by FastQC. Note that the background color indicates calls of very good quality in green, calls of reasonable quality in orange, and calls of poor quality in red.

PixelCut provides accurate predictions of low-quality truncation sites at the $3^{\prime }$ ends of reads by analyzing quality control (QC) reports generated by FastQC [14]. The predicted truncation positions can be used to guide paired-end read merging, thereby improving the overall accuracy of the analysis pipeline. Since QC reports are typically generated alongside sequencing results on platforms such as Illumina MiSeq and contain a rich combination of textual and visual information, we hypothesize that they can serve as a useful alternative to FASTQ files, which are the standard input for most 3′ end trimming tools. Furthermore, since QC reports are often used by researchers for manual interpretation during quality assessment, they can provide a straightforward way to validate the predicted trimming region. Motivated by this, we use the QC report generated by FastQC, especially the per-base sequence quality module, as the primary input for PixelCut, whereas other quality trimming tools generally use FASTQ files as input. Note that, when analyzing only the 16S rRNA region, non-biological nucleotides (e.g., primer and adapter sequences) should be removed before generating QC reports with FastQC, as these sequences may otherwise affect the quality distribution at the 3’ end. The per-base sequence quality report produced by FastQC is typically in HTML format and includes quality scores across all bases. It graphically represents the quality score of each base according to its sequencing position (Figure 1). First of all, the x-axis indicates the position of bases in the sequencing reads, and the y-axis shows the corresponding quality scores. Note that higher quality scores indicate more reliable base calls. For each position, bar plots with box-and-whisker plots show the distribution of quality scores. Specifically, for each bar, the yellow box shows the interquartile range, and the upper and lower whiskers indicate 10 and 90 percentiles, respectively. Additionally, the central red line in each bar indicates the median quality score, and the blue line across the *x*-axis represents the mean quality score across the x-axis. Note that the background colors indicate the quality of the reads. That is, green represents good quality, orange indicates moderate quality, and red signifies poor quality base calls.

### 2.1. Color Fragment Detection Algorithm

To accurately identify the trimming position at the end of each read, we analyze the pixel colors and textual captions in the per-base sequence quality report using the OpenCV library [15]. Since pixel-level color analysis can be computationally intensive, we employ a patch-based approach for color and character analysis.

Since the raw numeric values indicating the color intensity of the three channels (red, green, and blue) are not intuitive for human interpretation, we first construct a set of representative colors in the input image by analyzing image patches. Then, we construct a mapping table that connects fine-grained color values, such as light yellow and dark yellow, to broader color labels (e.g., yellow), which helps a more user-friendly classification of colors within the image. This mapping allows the system to generalize subtle color differences into interpretable categories, which is particularly useful when classifying regions based on perceived color types rather than raw pixel values. More specifically, we divide the input image into small, non-overlapping rectangular patches and collect the RGB pixel values from each patch. Note that the width and height of each rectangular patch are set to 10 pixels, as this size generally aligns well with the color index blocks in the per-base sequence quality image. Finally, we construct a color mapping from RGB pixel values to human-readable color labels.

Next, we identify the numeric character in the per-base sequence quality plot to determine the exact pixel location of the quality score threshold on the *y*-axis. Note that the default quality score threshold is set to 20, as it is widely used to ensure acceptable analytical results [16]. Additionally, the per-base sequence quality report highlights regions with quality scores below 20 in red, which corresponds to approximately a 1% error rate. This pixel location is then used as a reference point to search for the corresponding trimming position along the *x*-axis. Since the quality scores typically decrease as the *x*-axis increases, we search for the trimming position from right to left in order to reduce computational complexity. Starting from the right end of the image, we examine each image patch’s color. If the RGB pixel values can be classified as a broad yellow color, we continue the search. Otherwise, we stop the search, as it indicates the precise location where the quality scores fall below the threshold.

Once we identify the region where the background color transitions from yellow to another color (i.e., from the reliable-quality region to the low-quality region), we determine the exact trimming location using an OCR (Optical Character Recognition) function. Based on the identified color transition region, we extract image patches with a width of $w_{ocr}$ and a height of $h_{ocr}$ from the bottom of the per-base sequence quality plot. Note that we set $w_{ocr}$ to 80 and $h_{ocr}$ to 20, and we reduce the width of the image patch so that it aligns with the rightmost boundary of the image if the rightmost boundary extends beyond the per-base sequence quality plot. We utilize the image_to_string() function from the pytesseract library with specific language settings.

This process enables the system to robustly extract and interpret textual content from the dynamically selected region, which is later used to determine the current position or state of the image’s annotation region. Then, PixelCut reports the corresponding location in the per-base quality report as the potential trimming region.

### 2.2. Web Server Implementation

In order to enhance the usability of the proposed method and improve accessibility for those with limited computational resources, we implemented a cloud-based version of the trimming location prediction algorithm on a Linux system (Ubuntu 24.04). In order to provide a user-friendly web interface for the proposed pre-processing algorithm, we implemented the application using JavaServer Pages (JSP), deployed on an Apache Tomcat server (version 10.1.36)

On the front-end, we utilized the cos.jar library to enable multi-file input and file upload functionality directly in HTML. Uploaded input files, such as FastQC result HTML files, are then handled on the server side using the jakarta.servlet-api-6.0.0 package. To ensure session security, a unique directory is created based on the session ID, and the uploaded HTML files are stored and processed accordingly.

On the backend, we first extract the per-base sequence quality plots and the corresponding read cycle lengths from each HTML file. These extracted images and features are then used to analyze the quality plots and determine the truncation positions for read1 and read2. Finally, the proposed trimming location prediction algorithm identifies the optimal truncation positions for both reads based on the quality scores and returns the specific positions to the users.

**Figure 2:**
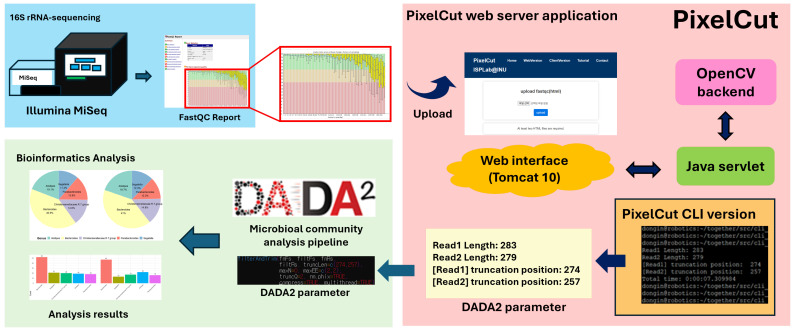
Example workflow of 16S rRNA sequencing analysis. PixelCut accepts a FastQC report and extracts quality score images for amplicon sequencing. It then automatically identifies the optimal trimming position at the $3^{\prime }$ ends of reads using the OpenCV library. This trimming position can subsequently be applied to downstream biological analyses within the 16S rRNA amplicon sequencing pipeline. Note that the region highlighted with a red bounding box represents the proposed method, while the remaining components illustrate a representative example workflow.

Note that although our main focus is the web-server implementation of the proposed method, we also release a standalone CLI (command-line interface) version so that those who want to incorporate the algorithm into their own pipelines can effectively integrate the proposed method. Figure 2 shows an overview of the web server implementation and an example workflow for analyzing 16S rRNA sequencing data.

## 3. Results

### 3.1. Dataset and Experimental Setup

To evaluate the effectiveness of PixelCut, our proposed web server application, we analyzed 16S rRNA amplicon sequencing data after trimming low-quality regions at the 3′ end of each read. We assessed the impact of 3′-end trimming using the DADA2 pipeline, which is widely employed for 16S rRNA amplicon sequencing analysis [9]. For this purpose, we first obtained publicly available 16S rRNA amplicon sequencing data from the European Nucleotide Archive (ENA) under accession numbers ERR3668534 and ERR3668535 [17]. The referenced study performed 16S rRNA sequencing on 40 human stool samples and achieved a total of 4,715,000 reads. This study compared taxonomic classification results to assess differences in gut microbial community composition. Since PixelCut automatically trims low-quality regions based on dataset-specific quality scores in the FastQC report, this adaptive mechanism effectively accommodates variability across datasets. Therefore, even with a limited number of datasets, the evaluation sufficiently captures the robustness of PixelCut under different read-length and quality-score patterns.

### 3.2. Evaluation of Microbiome Profiling Accuracy at Phylum and Genus Levels

Next, we compared the analysis results of PixelCut against FIGARO, which is one of the recommended 3′-end trimming methods in the official DADA2 tutorial [13]. To assess the impact of 3′-end trimming on analysis accuracy, we focused on taxonomic profiling at the phylum and genus levels using the DADA2 pipeline, following the guidelines provided in the official tutorial. In this benchmarking, we hypothesized that if PixelCut achieves a comparable level of taxonomic profiling performance to that of FIGARO, the developed web server application could serve as a user-friendly platform for both biologists and bioinformaticians, while also providing reliable analysis results.

**Figure 3:**
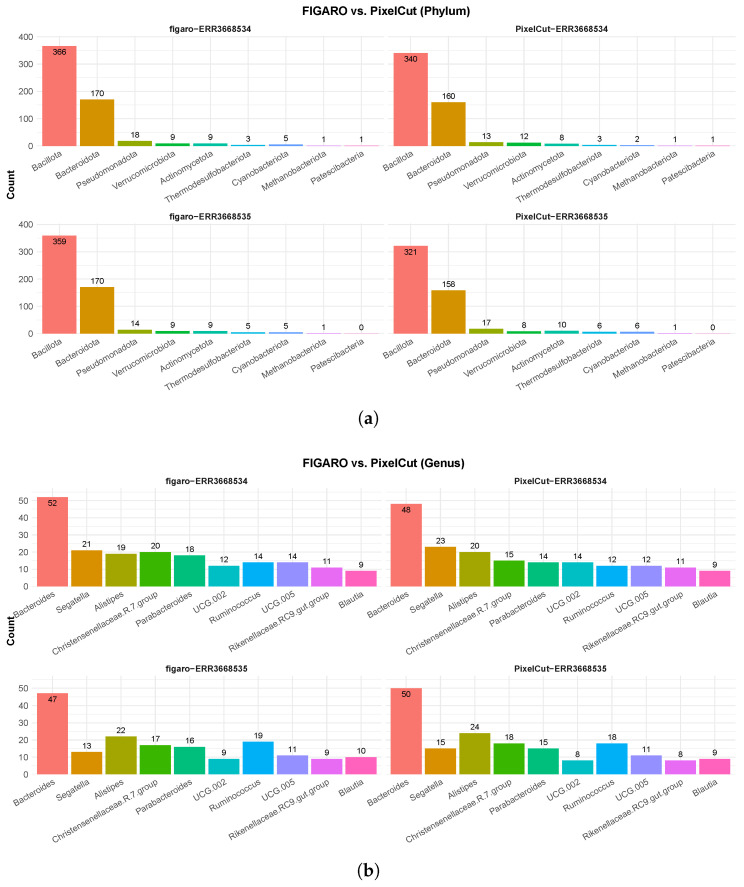
Benchmarking of taxonomic profiling on 16S rRNA amplicon sequencing data using FIGARO and PixelCut. (**a**) Taxonomic profiling results at the phylum level. Both FIGARO and PixelCut consistently identified dominant phyla, such as Bacteroidota and Firmicutes, with minor variations in low-abundance taxa. (**b**) Taxonomic profiling results at the genus level. PixelCut showed comparable profiling accuracy to FIGARO across both datasets.

We confirmed the taxonomic profiling at both the phylum and genus levels, and PixelCut and FIGARO showed similar profiling results (Figure 3). Although FIGARO detected more taxa at the phylum level, the differences were negligible, and both algorithms exhibited similar trends. Specifically, PixelCut and FIGARO consistently identified Bacillota and Bacteroidota as the dominant phyla, and the order of relative abundance for the dominant taxa followed the same pattern in both approaches. At the genus level, we observed a similar trend, with both methods consistently detecting Bacteroides, Alistipes, and Parabacteroides as the major genera. Moreover, although subtle differences were observed between the two approaches, the relative abundance and overall community structures were highly coherent. These results demonstrate that PixelCut achieves comparable performance to FIGARO in taxonomic profiling across multiple hierarchical levels, while also providing a web-based implementation that improves accessibility and reproducibility for researchers.

In addition to examining the detected counts at the phylum and genus levels, we also analyzed the proportional composition of taxa to confirm consistency between methods. We believe that relative proportions in taxonomic profiling provide more biologically meaningful insight than raw counts. That is, because sequencing depth and total library sizes often vary across samples or computational pipelines, direct count comparisons may introduce bias, whereas relative proportions inherently normalize for those differences and more reliably reflect the underlying community structure [18].

**Figure 4:**
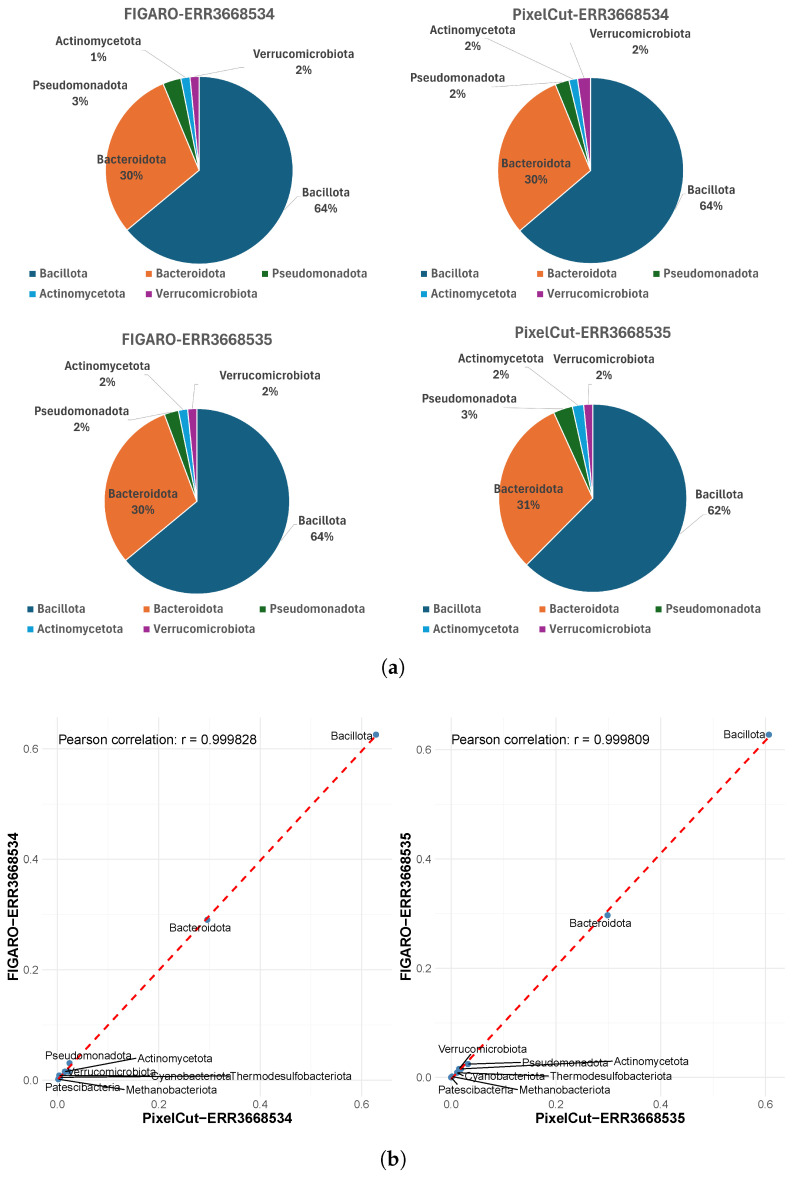
Comparison of taxonomic profiles at the phylum level between FIGARO and PixelCut. (**a**) Relative abundance of detected phyla visualized as pie charts for FIGARO and PixelCut. Both tools show similar phylum-level compositions, indicating consistent overall taxonomic proportions. (**b**) Correlation analysis of phylum-level abundances derived from FIGARO and PixelCut. Each point represents a phylum, and the strong Pearson correlation (r > 0.99) demonstrates high agreement between the two methods in taxonomic profiling.

**Figure 5:**
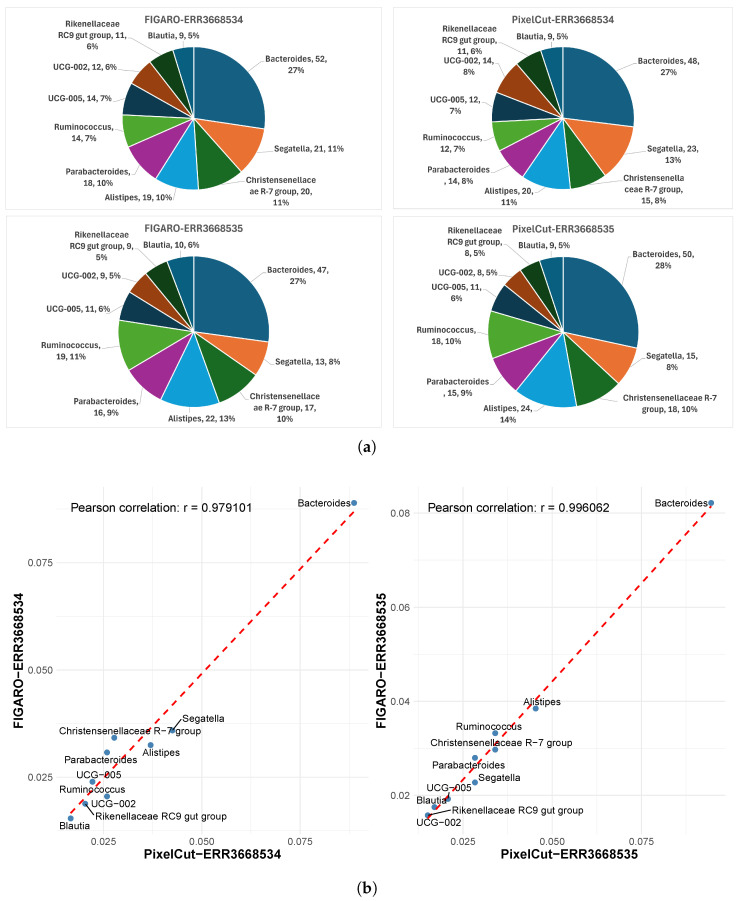
Comparison of taxonomic profiles at the genus level between FIGARO and PixelCut. (**a**) Relative abundance of detected genera visualized as pie charts for FIGARO and PixelCut. Both tools exhibit similar genus-level compositions, indicating consistent profiling of dominant taxa such as *Bacteroides* and *Ruminococcus*. (**b**) Correlation analysis of genus-level abundances derived from FIGARO and PixelCut. Each point represents a genus, and the high Pearson correlation (r > 0.97) demonstrates strong agreement between the two algorithms in taxonomic quantification.

First, although subtle differences exist in the number of detected phyla between the two methods, we confirmed that their relative proportions are almost identical (Figure 4). For instance, in sample ERR3668534, the proportions of the major phyla Bacillota and Bacteroidota are the same by both methods. Moreover, the correlation coefficient (Pearson’s r) of the proportions across phyla is 0.99, and the data lie very close to the reference line, indicating strong consistency between the methods. Similar to the phylum-level analysis, we also confirmed that the proportions of identified genera exhibit strong consistency (Figure 5), as evidenced by a high Pearson correlation. This consistent result implies that the impact of 3’-end trimming, whether via FIGARO or PixelCut, is negligible in terms of the biological interpretation of taxonomic profiles. Indeed, while absolute counts may differ slightly, the preservation of relative composition ensures that downstream ecological or comparative conclusions remain stable.

**Figure 6:**
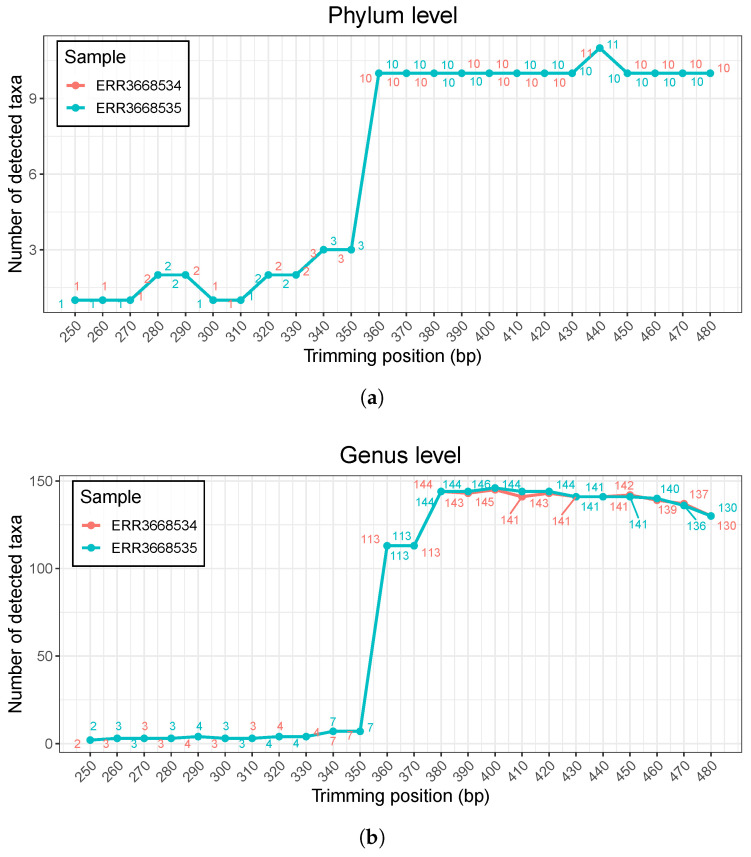
Sensitivity analysis of FIGARO with varying amplicon size parameters. (**a**) Number of detected taxa at the phylum level. The trimming positions determined by FIGARO under different amplicon size parameters were used in the DADA2 pipeline, and the resulting number of identified phyla is shown. (**b**) Number of detected taxa at the genus level. The genus-level results exhibit similar trends, where small changes in the amplicon size parameter lead to substantial fluctuations in the number of detected taxa.

We also conducted a sensitivity analysis of FIGARO by varying the assumed amplicon length, because amplicon length is a required user parameter for FIGARO, while our proposed method requires no hyperparameters. Since amplicon size is a required parameter to run FIGARO, one must estimate a putative amplicon size if the exact value is not available. To demonstrate the importance of accurate amplicon size estimation, we ran FIGARO with various assumed amplicon sizes (from 250 bp to 480 bp) and obtained trimming recommendations for each setting. Then, using the DADA2 pipeline, we examined how the number of detected taxa varied with the input amplicon length. Based on our benchmarking 16S rRNA sequencing datasets, the number of detected taxa (i.e., the count of taxa) changes dramatically depending on the provided amplicon length (Figure 6). When the estimated amplicon size is shorter than 350 bp, only a few phyla or genera are detected. From 360 bp onward, detection increases sharply. This suggests that using an incorrect amplicon size may lead to drastically different downstream results. This variation underscores the necessity of accurately estimating amplicon size to obtain reliable downstream results and clearly demonstrates that FIGARO’s trimming recommendations are highly sensitive to the input amplicon length. In contrast, because PixelCut does not require prior biological parameters such as exact amplicon size, it offers a more user-friendly and robust means of determining trimming positions that lead to stable downstream analyses. Therefore, while FIGARO’s reliance on an estimated amplicon length is a notable limitation and can pose a hurdle for users, our proposed method’s lack of need for strict prior specification provides a significant advantage by mitigating the risk of misestimating or misplacing trimming parameters.

### 3.3. Execution Time Evaluation

**Figure 7:**
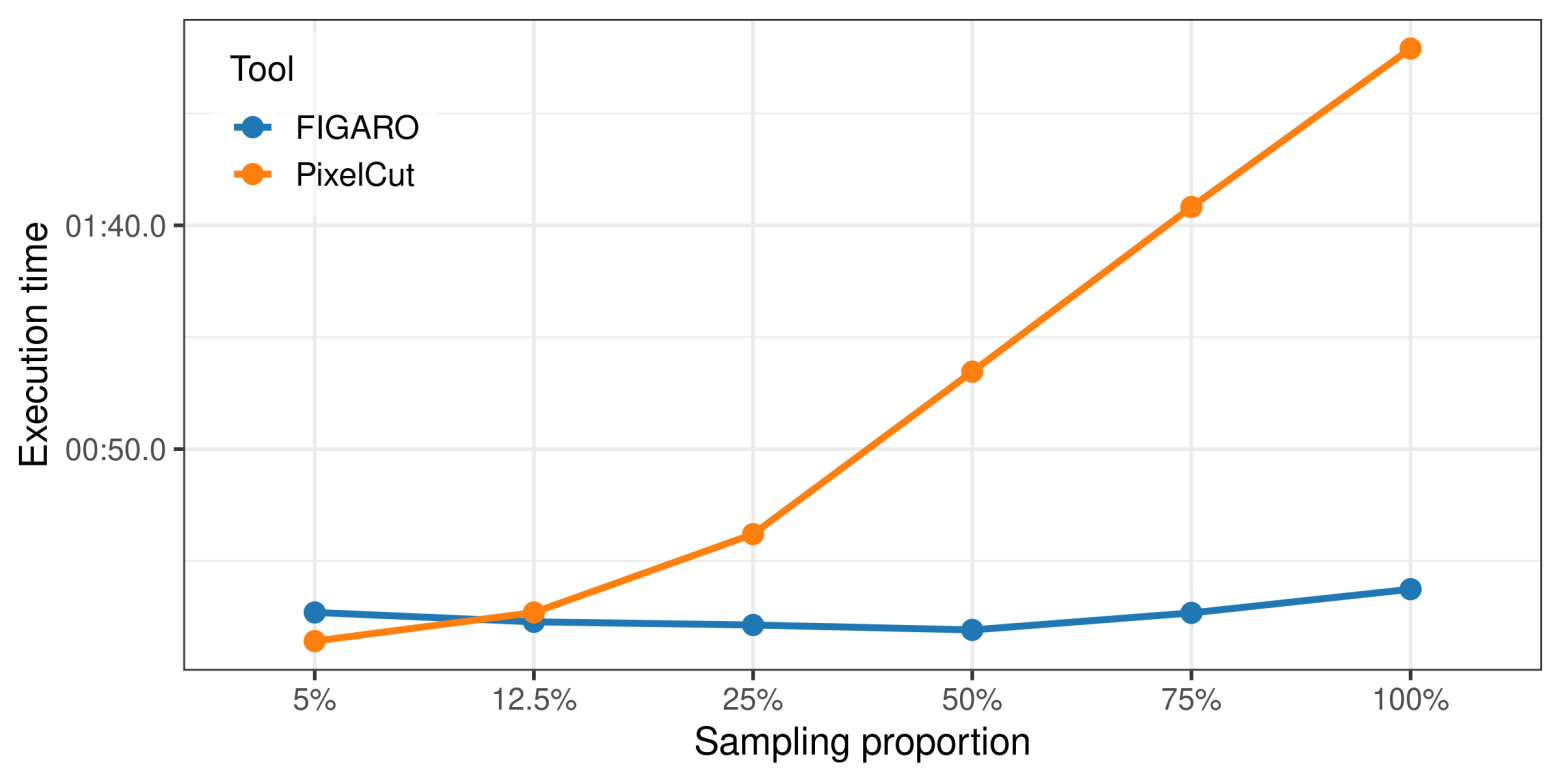
Comparison of runtime performance between FIGARO and PixelCut.

To evaluate the computational efficiency of PixelCut, we compared its execution time with FIGARO using benchmark datasets with different sampling ratios (Figure 7). Unlike FIGARO, which showed relatively stable performance regardless of the sampling proportion, PixelCut demonstrated a linear increase in execution time relative to the sampling proportion, primarily due to overhead from file reading during trimming. Specifically, when the sampling ratio was set to 5%, PixelCut (approx. 7.1 s) was approximately 2.6 times faster than FIGARO (approx. 18.7 s). However, FIGARO showed a faster runtime in other conditions. For the full dataset, FIGARO took approximately 13.4 s, while PixelCut took about 139.5 s. Although PixelCut requires slightly longer execution time, we consider the absolute duration to be practically acceptable, as microbiome data analysis pipelines commonly require tens of minutes to several hours of total processing time. Therefore, the difference in runtime is unlikely to be a limiting factor in real-world usage.

## 4. Discussion

In this work, we proposed a novel method called PixelCut for accurately predicting truncation positions at the 3′ ends of reads in 16S rRNA gene sequences. PixelCut leverages the per-base sequence quality report generated by FastQC, which is typically provided in HTML format. Using the OpenCV library, PixelCut analyzes the per-base quality plots embedded in the HTML report and predicts effective trimming locations to remove erroneous regions at the 3′ ends of reads. Through comparisons with FIGARO across multiple 16S rRNA datasets, we demonstrated that PixelCut achieves taxonomic profiling performance comparable to FIGARO at both the phylum and genus levels, while offering improved usability and reproducibility.

PixelCut has clear advantages over existing trimming-location prediction algorithms. While most trimming tools rely on the raw FASTQ file as input, PixelCut processes visual per-base quality plots directly, providing a more intuitive way to interpret results. Moreover, many pre-processing tools require users to supply hyperparameters, often demanding domain-specific knowledge or auxiliary experimental data. In contrast, PixelCut is fully automated and requires no user-defined parameters, helping ensure robust, consistent results. We also implemented the algorithm as a web-based application so that users without command-line expertise or advanced computing infrastructure can access it, which can lower the barrier to analyzing 16S rRNA sequencing data.

PixelCut employs a unified quality-score threshold that removes all nucleotides with quality scores below the specified cutoff across all input reads. This strategy may raise concerns that it could overlook per-read quality variations. However, from a practical perspective, Illumina MiSeq 16S amplicon sequencing typically exhibits quality degradation toward the 3’ end, and retaining low-quality bases can reduce the reliability of downstream taxonomic analysis [19]. Because high-quality regions generally maintain quality scores above Q20, while low-quality trailing regions consistently fall below Q20 regardless of per-read variability, the unified threshold results in balanced and effective trimming in practice. Moreover, since Q20 is a widely accepted standard cutoff for distinguishing unreliable regions, applying a unified threshold remains effective even in the presence of per-read quality differences [20]. Nevertheless, PixelCut provides the quality threshold as a tunable hyperparameter, allowing users to adjust the cutoff according to dataset-specific characteristics. Note that the selected quality threshold can influence both the length of the trimmed region and the confidence of downstream analyses. In general, aggressive trimming tends to reduce the number of detected taxa but increases the reliability of downstream results, whereas less aggressive trimming may produce a larger number of detected taxa but can also introduce less reliable taxa or potential false positives.

Although PixelCut provides a user-friendly, web-based application that accurately predicts trimming locations for 16S rRNA reads without additional user input, downstream analysis of trimmed reads must still be executed via separate workflows. In other words, PixelCut can only reduce the burden in the preprocessing stage, and there are still hurdles in the analysis pipeline for users without a sufficient software background to derive end-to-end taxonomic results. To lower these remaining hurdles, as future work, we plan to develop a fully integrated web platform that automatically performs downstream taxonomic and diversity analyses. Additionally, we will explore leveraging GPU acceleration or parallel computing to efficiently handle large-scale datasets in a web environment. Ultimately, this integrated future tool would make high-throughput 16S rRNA sequencing data analysis more accessible and less error-prone to users lacking bioinformatics expertise.

## Data Availability

The source code and a detailed user manual are freely available at https://github.com/eastbrain/PixelCut (accessed on 19 November 2025). The web application is accessible at http://robotics.inu.ac.kr/PixelCut (accessed on 19 November 2025). The raw sequencing data are deposited in the European Nucleotide Archive (ENA) under the accession number PRJEB35434.

## References

[1] Sanschagrin S., Yergeau E. (2014). Next-generation sequencing of 16S ribosomal RNA gene amplicons. J. Vis. Exp. JoVE.

[2] Clarridge J.E. (2004). Impact of 16S rRNA gene sequence analysis for identification of bacteria on clinical microbiology and infectious diseases. Clin. Microbiol. Rev..

[3] Seemann T. (2014). Prokka: Rapid prokaryotic genome annotation. Bioinformatics.

[4] Nurk S., Meleshko D., Korobeynikov A., Pevzner P.A. (2017). metaSPAdes: A new versatile metagenomic assembler. Genome Res..

[5] Lewis S., Nash A., Li Q., Ahn T.H. (2021). Comparison of 16S and whole genome dog microbiomes using machine learning. BioData Min..

[6] Janda J.M., Abbott S.L. (2007). 16S rRNA gene sequencing for bacterial identification in the diagnostic laboratory: Pluses, perils, and pitfalls. J. Clin. Microbiol..

[7] Caporaso J.G., Kuczynski J., Stombaugh J., Bittinger K., Bushman F.D., Costello E.K., Fierer N., Peña A.G., Goodrich J.K., Gordon J.I. (2010). QIIME allows analysis of high-throughput community sequencing data. Nat. Methods.

[8] Schloss P.D., Westcott S.L., Ryabin T., Hall J.R., Hartmann M., Hollister E.B., Lesniewski R.A., Oakley B.B., Parks D.H., Robinson C.J. (2009). Introducing mothur: Open-source, platform-independent, community-supported software for describing and comparing microbial communities. Appl. Environ. Microbiol..

[9] Callahan B.J., McMurdie P.J., Rosen M.J., Han A.W., Johnson A.J.A., Holmes S.P. (2016). DADA2: High-resolution sample inference from Illumina amplicon data. Nat. Methods.

[10] Callahan B.J., McMurdie P.J., Holmes S.P. (2017). Exact sequence variants should replace operational taxonomic units in marker-gene data analysis. ISME J..

[11] Del Fabbro C., Scalabrin S., Morgante M., Giorgi F.M. (2013). An extensive evaluation of read trimming effects on Illumina NGS data analysis. PLoS ONE.

[12] Prodan A., Tremaroli V., Brolin H., Zwinderman A.H., Nieuwdorp M., Levin E. (2020). Comparing bioinformatic pipelines for microbial 16S rRNA amplicon sequencing. PLoS ONE.

[13] Weinstein M.M., Prem A., Jin M., Tang S., Bhasin J.M. (2019). FIGARO: An efficient and objective tool for optimizing microbiome rRNA gene trimming parameters. bioRxiv.

[14] Bioinformatics B. (2010). FastQC: A Quality Control Tool for High Throughput Sequence Data. https://www.bioinformatics.babraham.ac.uk/projects/fastqc/.

[15] Bradski G., Kaehler A. (2008). Learning OpenCV: Computer Vision with the OpenCV Library.

[16] Ewing B., Hillier L., Wendl M.C., Green P. (1998). Base-calling of automated sequencer traces usingPhred. I. Accuracy assessment. Genome Res..

[17] Marizzoni M., Gurry T., Provasi S., Greub G., Lopizzo N., Ribaldi F., Festari C., Mazzelli M., Mombelli E., Salvatore M. (2020). Comparison of bioinformatics pipelines and operating systems for the analyses of 16S rRNA gene amplicon sequences in human fecal samples. Front. Microbiol..

[18] Weiss S., Xu Z.Z., Peddada S., Amir A., Bittinger K., Gonzalez A., Lozupone C., Zaneveld J.R., Vázquez-Baeza Y., Birmingham A. (2017). Normalization and microbial differential abundance strategies depend upon data characteristics. Microbiome.

[19] Whelan F.J., Surette M.G. (2017). A comprehensive evaluation of the sl1p pipeline for 16S rRNA gene sequencing analysis. Microbiome.

[20] Kioroglou D., Mas A., Portillo M.d.C. (2019). Evaluating the effect of QIIME balanced default parameters on metataxonomic analysis workflows with a mock community. Front. Microbiol..

